# Eruptive xanthoma as a warning sign of uncontrolled hypertriglyceridemia presenting with acute pancreatitis and uncontrolled type II diabetes mellitus: A case report

**DOI:** 10.1002/ccr3.8926

**Published:** 2024-05-22

**Authors:** Ankit Shrestha, Prabin Kumar Bam, Aakash Pandit, Hari Shrestha, Melisha Koirala

**Affiliations:** ^1^ Department of Internal Medicine Chitwan Medical College Bharatpur Nepal

**Keywords:** eruptive xanthoma, hypertriglyceridemia, pancreatitis, uncontrolled type 2 diabetes, xanthomatosis

## Abstract

**Key Clinical Message:**

Managing diabetic ketoacidosis (DKA) in individuals with severe dyslipidemia necessitates a comprehensive approach. While rehydration and continuous insulin infusion are fundamental components of DKA management due to the underlying insulin deficiency, the presence of severe hyperlipidemia with eruptive xanthomas warrants additional consideration. Early initiation of lipid‐lowering agents can expedite the resolution of cutaneous lesions and substantially mitigate the risk of severe complications such as pancreatitis, along with attenuating long‐term cardiovascular risks.

**Abstract:**

Xanthomas are the benign lesions which are generated by localized lipid deposits in the skin, tendons, and subcutaneous tissue. They appear clinically as yellowish papules, nodules, or plaques. Acute pancreatitis and eruptive xanthomas can occur as complications of hyperlipidemia. Uncontrolled diabetes mellitus in one of the risk factors for hypertriglyceridemia. Early recognition and treatment of the eruptive xanthomatosis as a warning sign of hypertriglyceridemia can decrease the morbidity and mortality due to acute pancreatitis. Here, we discuss a case of 37‐years old female patient with uncontrolled type II diabetes mellitus presented with acute pancreatitis and eruptive xanthomas as result of raised triglycerides and uncontrolled diabetes.

## 1 INTRODUCTION

Severe hypertriglyceridemia poses a potential life‐threatening risk for the development of acute pancreatitis, ranking as the third most common cause (1%–4%), while gallstones and chronic alcohol abuse remain the leading underlying factors.^1^ A deposit of lipids can form in the dermis (eruptive xanthomas) and in the retina (lipemia retinalis) in cases of severe hypertriglyceridemia with values greater than 2000 mg/dL. Despite the frequent descriptions of these symptoms in the medical literature, clinical observations of them are uncommon.^2^ Furthermore, serum triglyceride levels above 1000 mg/dL are a well‐known cause of acute pancreatitis, and in 50% of instances, they are linked with poorly controlled diabetes mellitus.^3^ We present a case of a patient with multiple yellowish, papules on the extremities suggestive of eruptive xanthomas admitted to our hospital with acute pancreatitis due to hypertriglyceridemia aggravated by poorly controlled type II diabetes mellitus.

## 2 CASE HISTORY/EXAMINATION

A 37‐year‐old female presented to emergency department of our hospital with the history of severe pain in the epigastric and left hypochondriac region for 1 day. Pain was followed by three episodes of non‐bilious vomiting containing food particles. Two weeks prior to this presentation she complained of diffuse pruritic rash on bilateral upper and lower limbs for which she had visited her local clinic where she was managed as an acute case of pruritic dermatitis. She denied of abdominal distension, joint pain, headache, fever, chest pain, cough, shortness of breath, and palpitation. Bowel habits and bladder functions were normal. She had a prior history of acute pancreatitis 18 months back during which she was diagnosed and treated for hypertriglyceridemia and acute onset diabetes mellitus.

Metformin and atorvastatin were started. In addition, she was counseled for dietary and lifestyle modification. But the patient had a sedentary lifestyle and was non‐adherent to diabetic dietary regimen. So, the patient presented with above symptoms.

On examinations (vitals were within normal limits), vitals showed temperature of 96.4 F, pulse rate of 94/min, respiratory rate of 20/min, blood pressure 120/70 mm Hg, saturating 98% on room air. Her body mass index was 25 kg/m^2^.

Epigastrium was tender with active guarding.

**FIGURE 1:**
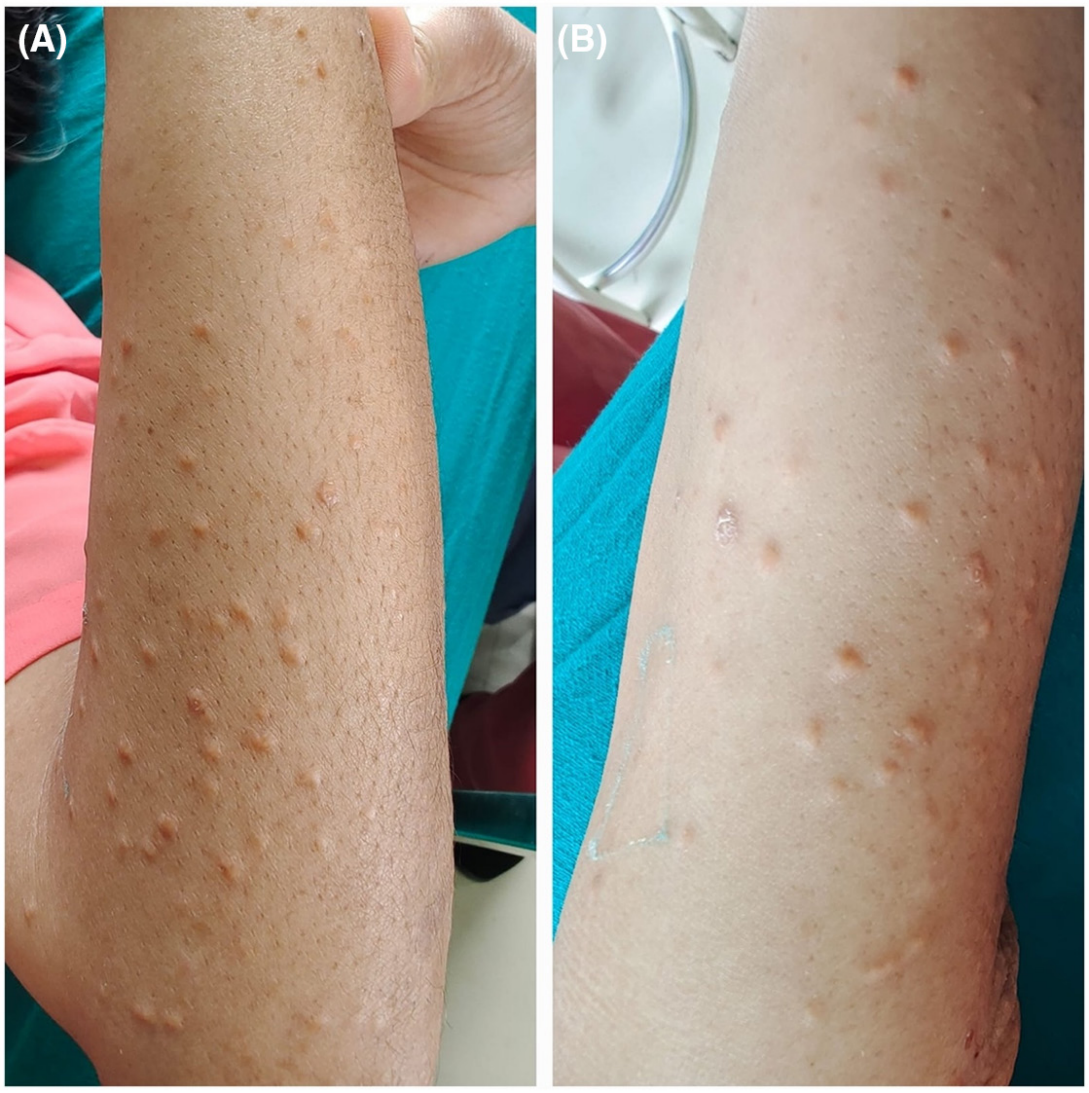
Xanthomatous eruptions on extensor surface of the arms (A); Xanthomatous eruptions on thigh (B).

Skin examination at the time of presentation demonstrated diffuse, multiple, painless, yellowish papules of various sizes (1–4 mm) distributed bilaterally on the skin of extensor surface of the arms and thigh with some involvement on the abdomen and trunk (Figure 1A,B).

There was no history of diabetes mellitus, hypertriglyceridemia, and similar type of lesions in the family.

## 3 METHODS (INVESTIGATIONS AND TREATMENT)

Blood drawn for investigations appeared lipemic with milky supernatant in the vacutainers.

Laboratory investigations revealed elevated serum lipase (2580 U/L), elevated HBA1C 10.3% and random blood glucose of 379 mg/dL.

Lipid profile of the patient showed elevated triglycerides (8480 mg/dL), total cholesterol 650 mg/dL, low‐density lipoprotein (LDL) 126 mg/dL, and high‐density lipoprotein (HDL) 90 mg/dL.

**FIGURE 2:**
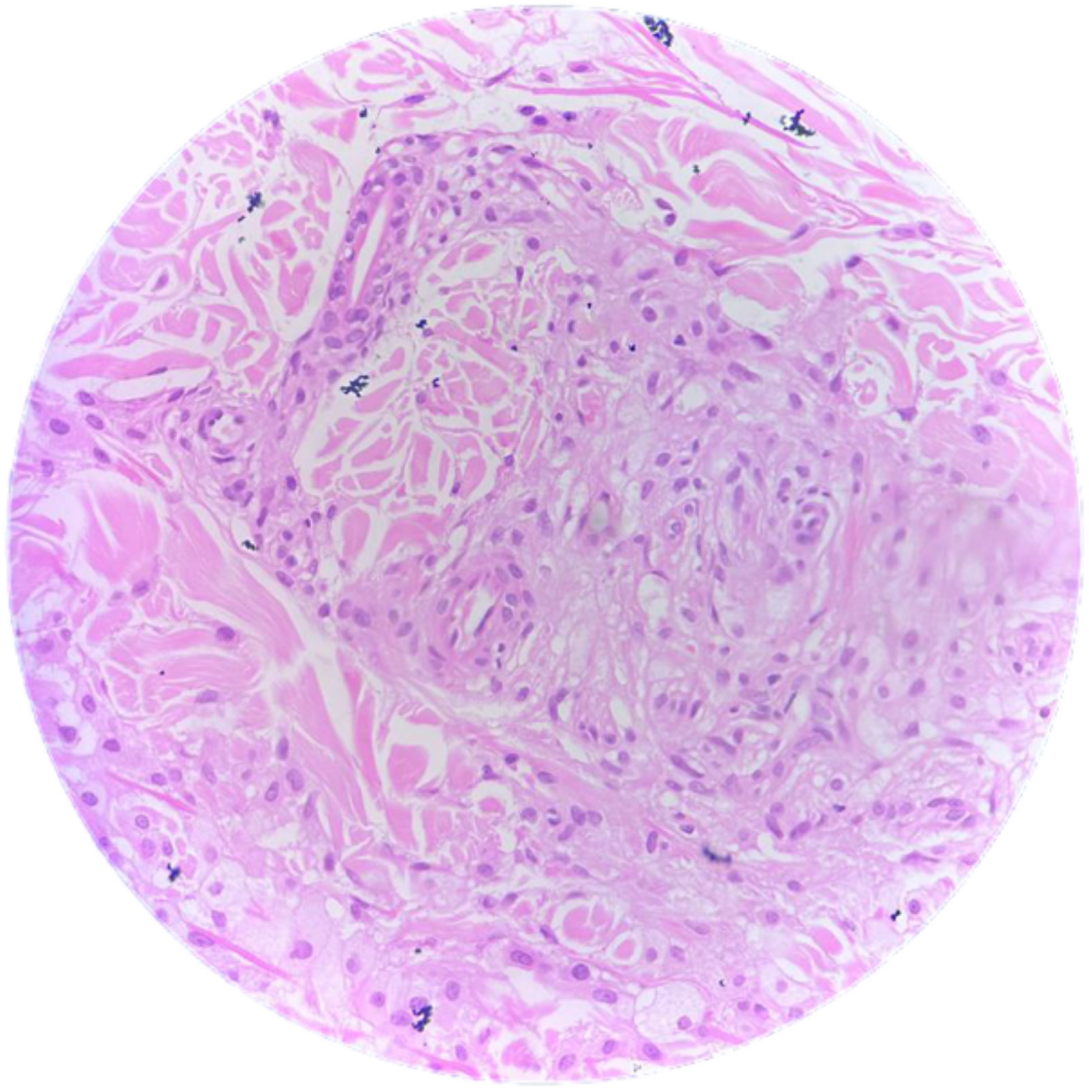
Microscopic image of the skin punch biopsy.

Skin punch biopsy showed fibro collagenous dermis with clusters of lipid laden foamy macrophages along with lymphoplasmacytic infiltration (Figure 2).

**FIGURE 3:**
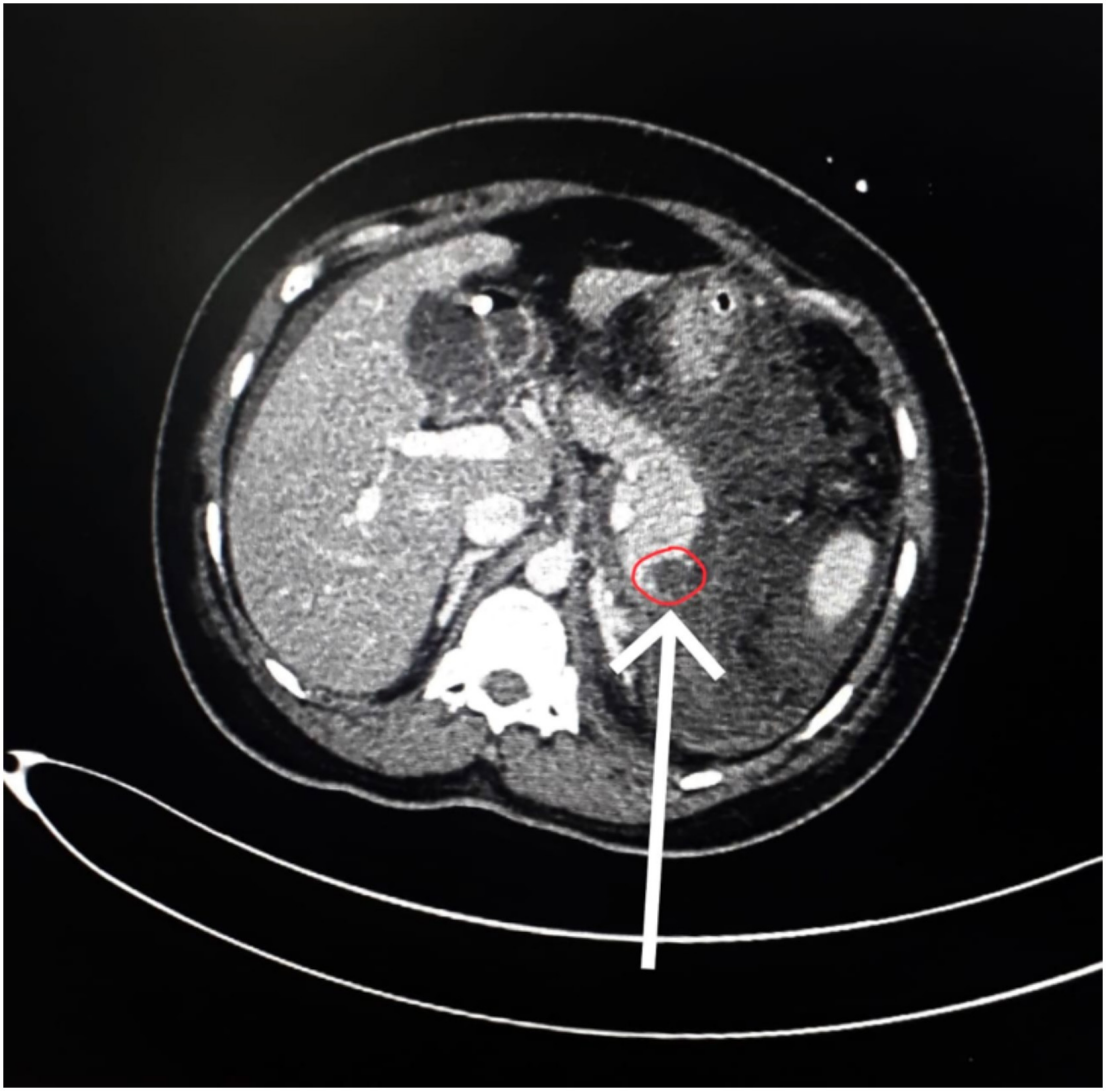
Arrow showing bulky pancreas with peripancreatic collection and non‐enhancing area around the tail suggestive of necrosis.

CT SCAN revealed bulky pancreas with fuzzy outline and heterogeneous attenuation and features suggestive of acute severe necrotizing pancreatitis (Figure 3).

Evaluation resulted in diagnosis of hypertriglyceridemia induced acute necrotizing pancreatitis and eruptive xanthoma due to poorly controlled diabetes mellitus.

Patient was treated with intravenous saline, analgesic, and insulin for 8 days. Pain and hyperglycemic symptoms subsided. She was subsequently treated with fibrates for hypertriglyceridemia and eruptive xanthoma. She was also advised with diabetic diet and lifestyle modifications.

## 4 CONCLUSION AND RESULTS (OUTCOME AND FOLLOW‐UP)

**FIGURE 4:**
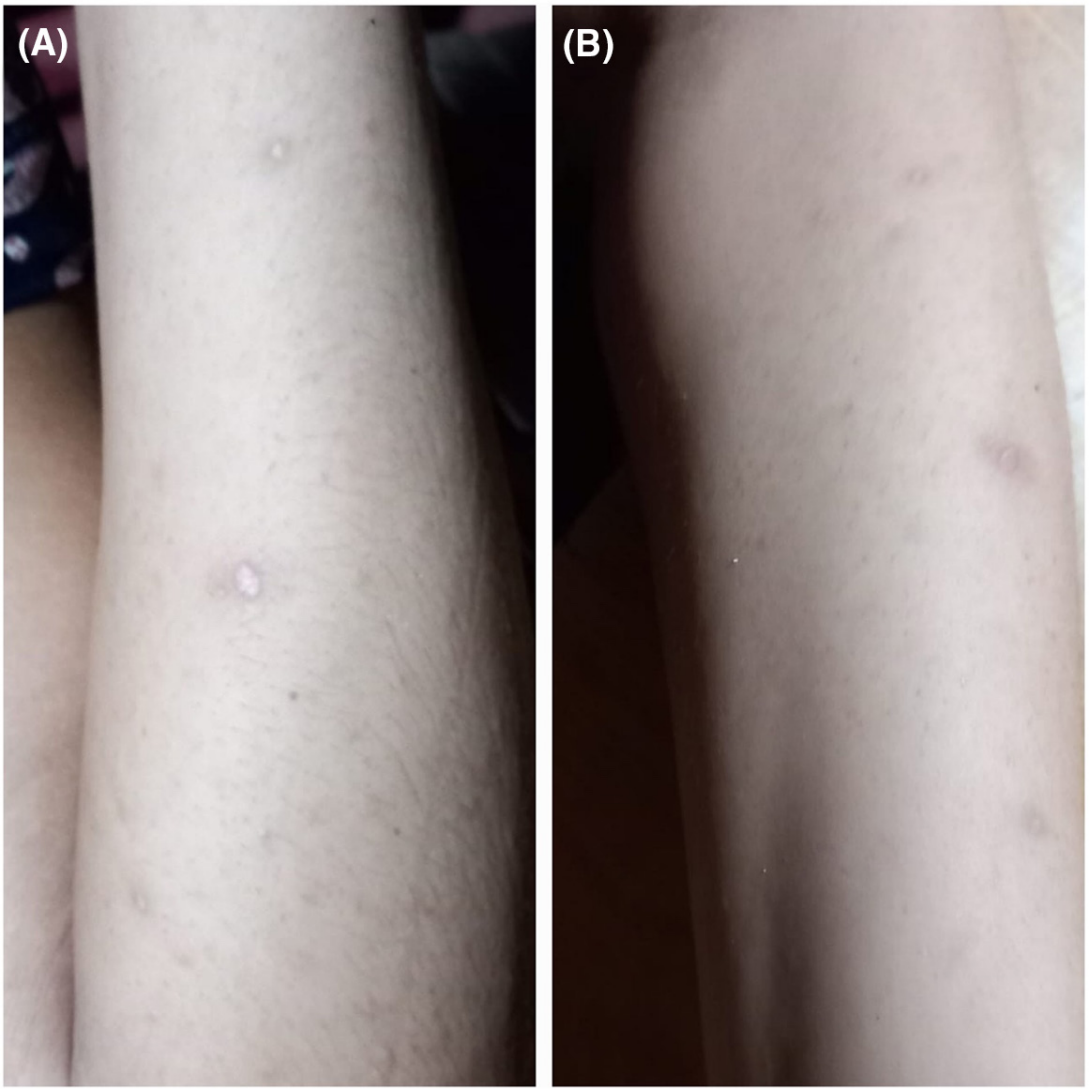
Resolved eruptive xanthoma after 30 days on extensor surface of the arm (A); Resolved eruptive xanthoma after 30 days on the thigh (B).

On her subsequent visits after 30 days triglycerides and total cholesterol levels had reduced to normal. Blood glucose level was within normal range. The eruptive xanthoma lesions had resolved as the hyperlipidemia improved (Figure 4).

## 5 DISCUSSION

Eruptive xanthomas are benign skin lesions that appear as sudden outbreaks of red or yellowish papules. They occur when lipids accumulate in the dermis due to high triglyceride levels, which leak through capillaries and are absorbed by dermal macrophages. The clinical presentations can vary from asymptomatic lesions to significant itching and sensitivity.^4^ In our case, 2 weeks prior to developing life threatening acute pancreatitis she had diffuse pruritic rash of xanthomatous lesions for which she visitied to her local clinic but was not investigated or treated for her underlying cause, which was uncontrolled hypertriglyceridemia. Xanthomas are identified histologically by the presence of extracellular lipid deposits and the growth of foam cells.^5^ They may arise in individuals with primary hypertriglyceridemia or secondary conditions like uncontrolled diabetes, alcohol abuse, or medication usage. Uncontrolled diabetes can contribute to elevated triglyceride levels, increased concentrations of VLDL and chylomicrons, and potentially trigger hypertriglyceridemia pancreatitis.^6^ Among the various causes of acute pancreatitis, biliary calculi, and alcohol intake account in 90% of the cases. Hypertriglyceridemia is an uncommon etiology (2%–10%).^7^ The pathogenesis of hypertriglyceridemia induced acute pancreatitis is still unknown. One theory is that large concentration of free fatty acids are generated through hydrolysis of triglycerides by pancreatic lipase which in turn induce pancreatitis.^8^ Hyperlipidemia induced acute pancreatitis should be managed similarly to other forms of acute pancreatitis. Presently, there is no substantial evidence suggesting that this type of pancreatitis differs from others in terms of necrosis frequency,^9^ complications or outcomes.^10^ To address the underlying hyperlipidemia, certain medicines, typically fibrates, are usually required.^11^ Managing diabetic ketoacidosis (DKA) in individuals with severe dyslipidemia necessitates a comprehensive approach. While rehydration and continuous insulin infusion are fundamental components of DKA management due to the underlying insulin deficiency, the presence of severe hyperlipidemia with eruptive xanthomas warrants additional consideration. Early initiation of lipid‐lowering agents can expedite the resolution of cutaneous lesions and substantially mitigate the risk of severe complications such as pancreatitis, along with attenuating long‐term cardiovascular risks.^13^

After treatment of underlying metabolic disorder, xanthomatous lesions mostly disappear without leaving scars. If the medical treatment fails to resolve the lesions surgery, laser or cryosurgery are therapeutic alternatives.^12^

Especially in patients with recently diagnosed or poorly controlled diabetes mellitus, the appearance of eruptive xanthomas should prompt assessment for severe hypertriglyceridemia. If the problem is not recognized, there is a higher chance of developing acute pancreatitis and other life‐threatening conditions. In order to provide adequate care, rigorous dietary restrictions, and HMG‐CoA reductase inhibitors must be used to manage the underlying hyperlipidemia.^4^

## 6 CONCLUSION

Early recognition of eruptive xanthomatosis as a warning sign for severe hypertriglyceridemia is crucial to prevent the potentially fatal complication of acute pancreatitis, enabling prompt diagnosis and tailored treatment to minimize associated complications. Additionally, a patient's wait time between seeing a doctor and starting treatment for a fatal medical condition can be shortened by being aware of the link between newly diagnosed or poorly managed diabetes mellitus and hypertriglyceridemia.

## AUTHOR CONTRIBUTIONS

**Ankit Shrestha:** Conceptualization; formal analysis; writing – original draft; writing – review and editing. **Prabin Kumar Bam:** Software; writing – review and editing. **Aakash Pandit:** Writing – review and editing. **Hari Shrestha:** Methodology; writing – review and editing. **Melisha Koirala:** Conceptualization; writing – original draft; writing – review and editing.

## FUNDING INFORMATION

All authors have declared that no financial support was received from any organization for the submitted work.

## CONFLICT OF INTEREST STATEMENT

The authors have no conflict of interest to declare.

## CONSENT

Written informed consent was obtained from the patient to publish this report in accordance with the journal's patient consent policy.

## Data Availability

Data available on request from the authors.
